# Supplementary material for: Effects of sports intervention on aggression in children and adolescents: a systematic review and meta-analysis
Source: PeerJ. 2023 Jun 13;11:e15504. doi: 10.7717/peerj.15504 (PMC10274581; doi:10.7717/peerj.15504)
Supplement: Supplemental Information 2 [file peerj-11-15504-s002.docx]

**The rationale for conducting the systematic review / meta-analysis**

**Answer:** Many studies have confirmed that sports intervention has a positive effect on aggressive behavior of children and adolescents. The higher the physical activity level of school-age children, the lower their aggressive behavior. However, not all studies have found a negative relationship between sports and adolescent violence. Mendez, Ruiz-Esteban and Ortega (2019) indicated that students who practiced physical activity at least four or more times per week, had higher values in the indicators of aggressiveness than students who practiced less frequently. Michael et al. reported that some rough physical contact in sports, or even fighting, actually leads to an increase in adolescent aggression. This systematic review aims to integrate the existing research on sports intervention and explore the impact of exercise on children and adolescents' aggression. According to existing research, analyze whether different conditions in the intervention, such as type of sports, intervention duration, have different influence on the effect of interventions.

**The contribution that it makes to knowledge in light of previously published related reports, including other meta-analyses and systematic reviews**

**Answer:** Spruit et al. conducted a meta-analysis reporting that there was no overall significant association between sports participation and juvenile delinquency, sports participation could not reduce the occurrence of juvenile delinquency. Our review aimed to explore the impact of sports intervention on aggression and other externalizing behaviors of aggression, including hostility, anger, delinquent acts, attitude towards violence (ATV) and provocation/bullying.
